# NanoARPES of twisted bilayer graphene on SiC: absence of velocity renormalization for small angles

**DOI:** 10.1038/srep27261

**Published:** 2016-06-06

**Authors:** I. Razado-Colambo, J. Avila, J.-P. Nys, C. Chen, X. Wallart, M.-C. Asensio, D. Vignaud

**Affiliations:** 1 I.E.M.N., UMR CNRS 8520, Av. Poincaré CS 60069, 59652, Villeneuve d’Ascq Cedex, France; 2 Synchrotron SOLEIL, L’Orme des Merisiers, Saint Aubin-BP 48, 91192 Gif sur Yvette Cedex, France

## Abstract

The structural and electronic properties of twisted bilayer graphene (TBG) on SiC(000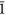) grown by Si flux-assisted molecular beam epitaxy were investigated using scanning tunneling microscopy (STM) and angle-resolved photoelectron spectroscopy with nanometric spatial resolution. STM images revealed a wide distribution of twist angles between the two graphene layers. The electronic structure recorded in single TBG grains showed two closely-spaced Dirac π bands associated to the two stacked layers with respective twist angles in the range 1–3°. The renormalization of velocity predicted in previous theoretical calculations for small twist angles was not observed.

Twisted bilayer graphene (TBG) became a subject of intense studies because of its rich physical and electronic properties. In TBG, two layers are stacked with a variable twist angle between them. For large twist angles, the two graphene layers are electronically decoupled such that close to the Dirac point the electronic properties are identical to that of single layer graphene^1^. Theoretical^2^,^3^,^4^,^5^,^6^,^7^,^8^,^9^,^10^,^11^,^12^,^13^,^14^ studies showed that varying the twist angle between the layers results into a change in the interlayer interaction especially for low angles. This leads to new electronic properties such as renormalization of Fermi velocity *v*_*f*_^2^,^3^ and occurrence of van Hove singularities^8^,^9^,^10^,^11^,^12^,^13^,^14^,^15^. These open the possibility of tuning the electronic properties by changing the twist angle^16^,^17^. Using the continuum approximation^2^, it was found that Dirac cones with single layer behavior still exist in bilayer graphene, but with a significant reduction of *v*_*f*_ for small angles.

On the experimental side, serious inconsistencies still remain on *v*_*f*_ renormalization for small angles. Many studies^18^,^19^,^20^,^21^ on twisted graphene performed using combined scanning tunneling microscopy/spectroscopy (STM/STS) and Landau-level spectroscopy (LLS) revealed *v*_*f*_ renormalization. For instance, transferred graphene layers grown by chemical vapor deposition (CVD) with a 3.5° twist angle revealed a *v*_*f*_ of 0.87 × 10^6^ m/s^20^. Another LLS study that showed reduced *v*_*f*_ (0.82 × 10^6 ^m/s) for 3.1° twist angle involved few layer graphene on highly-oriented pyrolitic graphite (HOPG)^21^. On the contrary, LLS^22^, STM/STS^15^ and angle-resolved photoelectron spectroscopy (ARPES)^23^ studies of twisted graphene multilayers on graphene/SiC (C-face) did not reveal any reduction in *v*_*f*_ compared to single-layer graphene. Another STM/STS study^24^ on CVD grown TBG on Rh foil did not exhibit reduction in *v*_*f*_ for twist angles between 1° and 3°. ARPES^23^ experiments also showed a clear crossing of the Dirac cones instead of a hybridization-induced anti-crossing even for small twist angles. A *μ*m lateral resolution ARPES study was recently reported^25^, where *v*_*f*_ renormalization was observed at 2.7° twist angle. However, it is not clear if the signal shown in the *μ*ARPES images came from adjacent or stacked grains. The main drawback of these ARPES studies on multiple graphene grains is that the photoelectron intensity may originate from adjacent grains rather than from a single grain due to the large incident beam. So far, *v*_*f*_ renormalization on twisted graphene remains an issue, which could be linked to the structure-dependent TBG electronic properties associated with the different elaboration techniques.

In this work, we address the electronic structure of single grains of TBG on SiC(000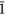) by using high energy and angular resolution ARPES, with nanometric lateral resolution (nanoARPES). The novelty of this work lies in the direct measurement of the band structure within individual TBG grains with small twist angles, thus leading to an accurate determination of band velocity. These experiments were achieved in exactly 2 monolayer (ML)-thick grains, calibrated by simultaneous core-level and ARPES measurements with nanometric lateral resolution^26^. Because the photon spot size was much smaller than the TBG grains, we were able to measure the band structure close to the Dirac point of single TBG grains for small twist angles: no velocity renormalization occurred at 2.1° and 1.8° ± 0.6° twist angles.

## Results

**Figure 1:**
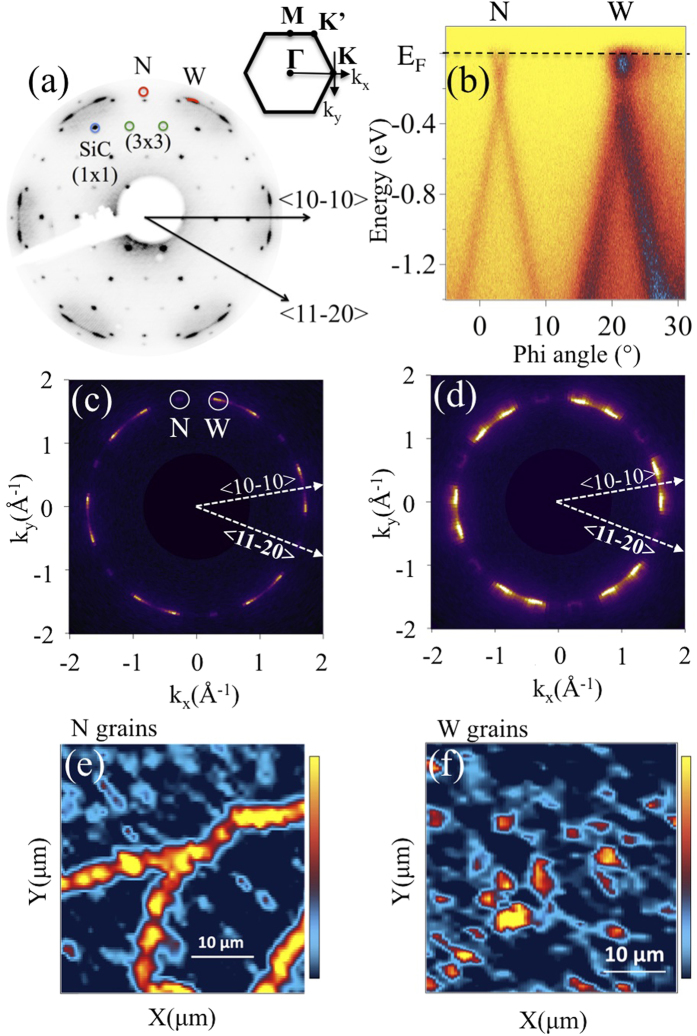
LEED pattern at 77 eV electron energy showing the graphene [N (red hollow circle) and W (red curve) azimuths], (3 × 3) (green hollow circles) and SiC (1 × 1) (blue hollow circle) diffraction spots, (inset) graphene Brillouin zone showing the high symmetry directions (**b**) Dirac cones N and W measured perpendicular to the Γ − *K* direction, obtained at 100 eV photon energy; ARPES constant energy maps (**c**) at the Fermi level and (**d**) at 1.0 eV below the Fermi level; nanoARPES intensity real-space maps (40 × 40 *μ*m^2^) measured along the respective Γ − *K* directions of (**e**) N- and (**f**) W- type grains. The intensity originates from the integration of the *π* states covering the energy range from 0 to 0.5 eV below the Fermi level [note: (**e**,**f**) obtained at different areas of the sample]. Panels (b–d) were recorded using conventional ARPES (spot size 90 *μ*m).

Graphene single grains on SiC(000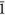) are oriented along two preferred azimuths^26^,^27^,^28^,^29^. The low energy electron diffraction (LEED) pattern in Fig. 1(a) shows a narrow spot labelled N, aligned along the <11
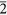
0> SiC directions, and a wider spot labelled W, approximately located ±3–11° on both sides from the <10
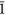
0>. The ARPES constant energy maps in Fig. 1(c,d), respectively measured at the Fermi level and at 1.0 eV below the Fermi level, show the same graphene pattern as the one observed by LEED [Fig. 1(a)]. The band structure including the Dirac cones oriented along the N and W directions is shown in Fig. 1(b) as a function of the azimuthal angle. It corresponds to a cut through the *K* point (at *k*_||_ = 1.703 Å^−1^) perpendicular to the Γ − *K* direction [see inset of Fig. 1(a)] of Dirac cones oriented along different crystallographic directions. Indeed, the N cone is quite sharp while the W cone has a wider azimuthal distribution. The N and W bandwidths are large, which may originate from nearly aligned different grains considering that the beam size is larger than the grain size. The N width is 0.06 Å^−1^ 26, and the W width is even larger, because its continuous orientation distribution is much wider. To overcome this limitation, we have utilized the state-of-the-art high spatial resolution scanning photoemission k-microscope at the ANTARES beamline. NanoARPES intensity real-space maps for the N and W grains are depicted in Fig. 1(e,f) respectively to emphasize the grain size and distribution on the surface. This was done by measuring the photoelectron intensity of the *π* Dirac bands at the *K* point, along the corresponding Γ − *K* direction. Note, however, that imaging the same area for both azimuths is only possible if it is exactly at the center of rotation of the microscope. The N grains in Fig. 1(e) show a rather inhomogeneous grain distribution as exemplified by the high intensity lines, while the W grain distribution in Fig. 1(f) is less organized. Consequently, using the point mode acquisition, we could measure the band structure within any single N or W grain. After correlated nanoARPES and nano core-level experiments^26^, we showed that N grains only involve AB stacked multilayers (typically 4–6 ML, see supplementary information Fig. S1) while W grains mainly include TBG. The electronic structure of AB stacked layers is already well established as compared to the more controversial twisted configuration. From this point, we focus our attention on the W grains associated with TBG.

The twist angle *θ* between graphene layers can be directly imaged in real space by STM as manifested by the occurrence of large-period superlattices known as moiré patterns^15^,^27^,^30^. The moiré periodicity *D* depends on *θ* and the graphene lattice constant *a*^31^,


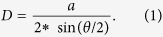


**Figure 2:**
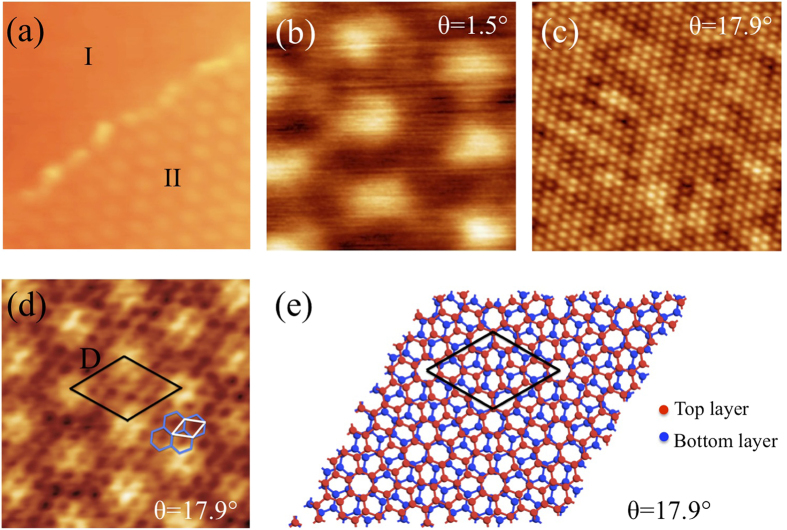
STM images of bilayer samples, (20 × 20 nm^2^) showing (**a**) non-moiré (I) and moiré (II) (7.3° twist angle) areas (−1 V sample bias, 50 pA tunneling current); moiré patterns with twist angles of (**b**) 1.5° and (**c**) 17.9°, (**d**) (3 × 3 nm^2^) STM image zoom of (**c**) showing the moiré superlattice (black unit cell), the graphene lattice (white unit cell) and the graphene honeycomb structure (blue), [(**b**–**d**) −2 V sample bias, 100 pA tunneling current], (**e**) scheme of the moiré in (**d**).

As *θ* decreases, the size of the moiré cell enlarges. Our STM studies revealed the presence of areas without (I) or with (II) moiré patterns as shown in Fig. 2(a). The non-observation of a superstructure in bilayer graphene might be explained either by the occurrence of AB stacking^27^ or by a large twist angle, resulting in a moiré with a very low amplitude^31^. Figure 2(b,c) are representative STM images revealing moiré superstructures of the bilayer samples showing the maximum and minimum periodicities, respectively 9.6 and 0.79 nm. From Eq. (1), the corresponding twist angles are calculated to be 1.5° and 17.9°. A wide range of moiré periodicities was observed which implies a wide distribution of twist angles between graphene layers. Figure 2(d) is a zoom of Fig. 2(c) showing both the moiré superlattice (black cell) and the 1 × 1 graphene lattice (white cell). The honeycomb structure (marked blue) of the surface graphene layer is observed, which was reported^27^ to imply monolayer behavior of the twisted graphene layers. Figure 2(e) is the atomic structure for a 17.9° twist angle and shows a perfect fit with the experimental moiré in Fig. 2(d).

**Figure 3:**
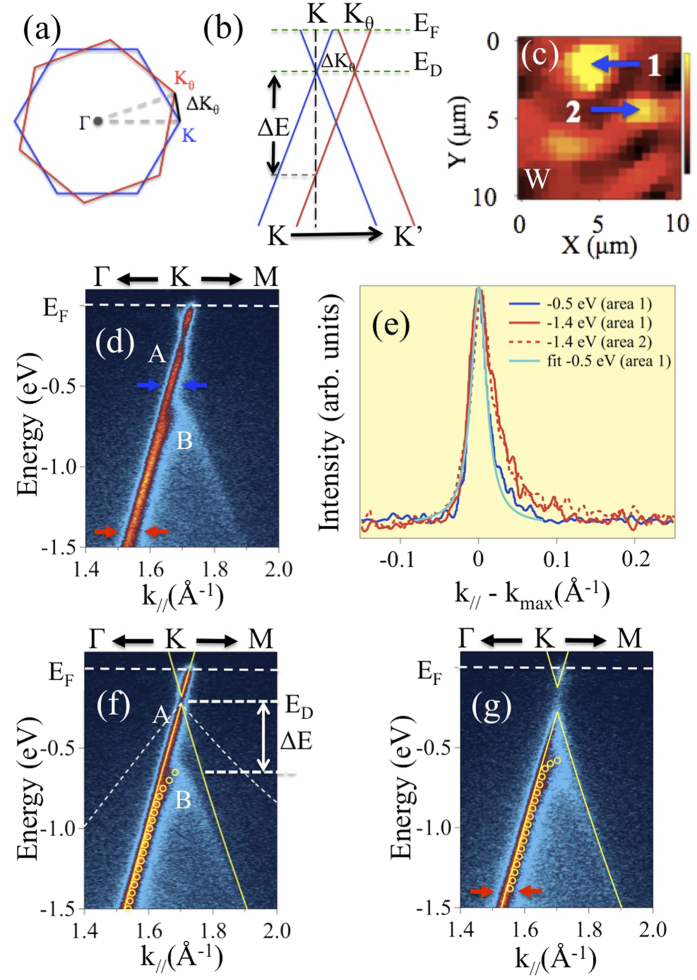
Schematics of the (**a**) Brillouin zones and (**b**) corresponding Dirac cones for two graphene layers twisted by an angle *θ*; (**c**) nanoARPES intensity real-space map (10 × 10 *μ*m^2^) measured along the cone W direction. The intensity originates from the integration of the *π* states covering the energy range from 0 to 0.5 eV below the Fermi level; (**d**) E(**k**) dispersion measured parallel to the Γ − *K* direction at grain 1 as marked in (**c**); (**e**) intensity line profiles from the MDCs as indicated by the arrows in (**d**) at energies −0.5 eV (blue) and −1.4 eV (red) and in (**g**) at energy −1.4 eV (red dotted curve) (all MDCs were horizontally shifted to present an intensity maximum at k_||_ − k_max_ = 0); E(k) dispersions at region 1 (**f**) and 2 (**g**) [see panel (c)], with superposed tight-binding fit (band A) and yellow hollow circles deduced from the experimental maximum intensities of the MDCs (band B), 100 eV photon energy [note that the same measurement appears in panels (d,f)]. The white dashed lines drawn in panel f shows the expected renormalized band dispersion for *θ* = 2.1°.

When two graphene sheets are superposed with a relative twist, the corresponding BZs also rotate with respect to each other as illustrated in Fig. 3(a). The twisted Dirac cones associated to each graphene layer are schematized in Fig. 3(b). Both sides of each cone are visible when measuring along the *K* − *K*′ direction, while only one side of each Dirac cone is observed when probing along the Γ − *K* direction, due to matrix element effects^32^. The latter experimental geometry applies to our nanoARPES experiments. Measurements perpendicular to the Γ − *K* direction [see Fig. 3(a)] imply azimuthally rotating the sample which is only possible if the grain of interest is exactly at the center of rotation of the microscope. This requirement is usually not met on samples with small grains (few *μ*m^2^ size). The electronic properties of TBG single grains were explored on a sample with a nominal thickness of 1.6 ML by nanoARPES^33^. This 1.6 ML sample is well-suited for electronic band structure studies of TBG since most of the surface is covered by bilayers, as previously shown on the same sample^26^. Figure 3(c) is a nanoARPES intensity real-space map recorded at the *K* point of the graphene BZ for grains oriented along the W azimuth. These grains mainly contribute to the photoelectron intensity. However, because of the large angular distribution of the W orientation, graphene grains which are slightly misaligned with respect to the set azimuth also contribute to the signal. These experimental conditions also imply the observation of an apparent band gap when the measured grain and the set azimuth are not perfectly aligned^34^. Two grains with the highest photoelectron intensities (yellow) are labelled 1 and 2. The grain 1 size is ~2 × 2 *μ*m^2^ which is much larger than the beam size (~120 nm). With the ANTARES nanoARPES set-up, individual TBG grains are probed, thus eliminating possible contributions from adjacent grains in the photoelectron intensity.

Using the microscope point mode, the nanoARPES E(k) dispersion was measured along the Γ − *K* direction for grain 1. The corresponding measurement is shown in Fig. 3(d), which suggests that two linear *π* bands are detected. The band A involves states up to the Fermi level *E*_*F*_, while the other one labelled B only appears for energies below ~ −0.65 eV. The occurrence of two bands was checked by performing a Lorentzian fitting analysis of the full width at half maximum (FWHM) of the experimental momentum distribution curves (MDC) [see Fig. 3(e)]. At −0.5 eV, the FWHM is 0.024 Å^−1^ which is the typical value for a single *π* band^35^. It is significantly narrower than the FWHM (0.060 Å^−1^)^26^ measured in conventional ARPES (with a ~90 *μ*m beam), where numerous grains contribute to the photoelectron intensity. This narrow FWHM validates the high spatial resolution capability of the k-microscope, such that single TBG grains could be probed. A larger width of 0.037 Å^−1^ is obtained at −1.4 eV, which is consistent with the occurrence of two bands appearing as one wider single band because their separation is lower than their FWHM. The fitted curve (light blue) for the MDC at −0.5 eV is superposed in Fig. 3(e). It also implies that a second component is required to fit the MDC at −1.4 eV. Tight-binding fitting for band A using the graphene monolayer equation^36^ is superposed to the experimental E(k) dispersion in Fig. 3(f). The corresponding Dirac point lies 0.2 eV below *E*_*F*_ and the band velocity deduced from the slope *dE*/*dk* close to the Dirac point is (1.1 ± 0.03) × 10^6^ m/s, which is typical of monolayer graphene. For band B, yellow hollow circles deduced from the experimental maximum intensities of the MDCs are superposed as guides to the eye. These observations show that two bands are present in the E(k) dispersion in region 1. Band A is associated to the graphene layer oriented very close to the Γ − *K* direction of the set azimuth in the electron analyzer [analogous to the blue Dirac cone in Fig. 3(b)]. The second band not reaching the Fermi level originates from the other graphene layer that is twisted with respect to the Γ − *K* direction of the corresponding azimuth [analogous to the red Dirac cone in Fig. 3(b)]. The maximum energy where band B is observed is illustrated in Fig. 3(b) as the intersection between the twisted band (red cone) and the Γ − *K* direction (black dashed line). Figure 3(g) is the band structure at region 2 also showing two bands as evidenced by the measured FWHM at −1.4 eV (red arrows) which is 0.036 Å^−1^. The velocity is determined to be (1.06 ± 0.05) × 10^6^ m/s for region 2.

The twist angle between the graphene layers can be quantified from the relative energetic positions of the two bands. In TBG, the Dirac cones are also rotated by the same angle *θ* in momentum space. They are centered at K and K_*θ*_, as illustrated in Fig. 3(b), and separated by^20^,


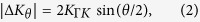


where *K*_Γ*K*_ = 4*π*/3a. As shown in Fig. 3(f,g), the band structure for both bands in regions 1 and 2 follows a linear relationship,


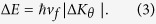


The Δ*E* value extracted from the two Dirac cones is ~0.45 eV, which leads to a Δ*K*_*θ*_ value of 0.062 Å^−1^ for the *E vs. k* plot in Fig. 3(f). The resulting twist angle between the two graphene layers based on Eq. (2) is ~2.1°. Theoretical calculations predict^8^ that at this small twist angle, *v*_*f*_ should be reduced to ~35% of the monolayer value (case n = 15, m = 16)^8^. The calculated renormalized band is drawn in Fig. 3(f) for comparison. Clearly, the renormalization is not observed in our experimental results as band velocity (1.1 × 10^6^ m/s) remains very similar to that of monolayer graphene. For the E(k) dispersion shown in Fig. 3(g), both cones do not reach the Dirac point implying that both graphene layers are twisted relative to the Γ − *K* direction of the set azimuth. The energy maxima (relative to *E*_*F*_) for which the two bands are detected are respectively 0.58 and 0.32 eV. Using the same arguments and equations as above, the corresponding twist angles are 1.8° and 0.6° relative to a virtual graphene layer aligned along the set azimuth. Since the nanoARPES experiments do not give information on the relative angular position of the graphene layers, the corresponding twist angle is 1.8° ± 0.6° (see supplementary information Fig. S2). The calculated renormalization^8^ is ~0.4 for 2.4° and ~0.05 for 1.2°. As mentioned above, the velocity obtained at region 2 is (1.06 ± 0.05) × 10^6^ m/s, inconsistent with any renormalization at this small twist angle. The twist angle range detected by STM (1.5°–17.9°) appears larger than the one obtained by nanoARPES (~1–3°). Because of the energy range which was used, twist angles larger than ~4° could not be detected by nanoARPES (see supplementary information, Fig. S3). This limitation directly comes from the tradeoff between experimental duration and energy range inherent to nanoARPES, and cannot be taken as an indication that the samples studied by STM and nanoARPES had different structures.

## Discussion

The above findings clearly indicate that the predicted *v*_*f*_ renormalization is not observed in our experiments for small twist angles. This is consistent with several experimental studies on the SiC C-face^15^,^22^,^23^ and on other twisted graphene material systems^21^,^24^. In ref. 15, *v*_*f*_ was extracted from the energy separation of the van Hove singularities in different grains with twist angles in the range 1°–10°. The calculated *v*_*f*_ based on the continuum model was 1.12 × 10^6 ^m/s in perfect agreement with our results. ARPES is a powerful tool to directly measure the band dispersion and clearly resolve the Dirac cones, thus can unambiguously and accurately determine *v*_*f*_. The ARPES work in ref. 23 did not detect any *v*_*f*_ reduction for twist angles as low as 1.1°, using conventional ARPES (tens of *μ*ms beam size) on multilayer samples. By measuring directly the band dispersion in single TBG grains, thanks to the high spatial resolution of the nanoARPES set-up, our results clearly evidence the absence of velocity renormalization for TBG on the C-face of SiC. Some previous works addressing this issue were performed using STS^15^,^16^,^17^,^18^,^19^,^20^,^21^,^22^,^23^,^24^, but required measurement of the energy difference of the van Hove singularities over different grains to extract *v*_*f*_. Based on our results obtained for bilayer grains and on previous results for multilayers, one can conclude that no renormalization takes place in the twisted graphene on the SiC C-face, regardless of the graphene thickness. This imply that the graphene-graphene interaction in this system is almost negligible. On the other hand, the interaction of twisted graphene layers with metallic substrates^19^,^20^ or HOPG^19^,^20^,^21^ could play a role in the reduction of *v*_*f*_ as discussed below.

One experimental proof of *v*_*f*_ renormalization is based on the LLS study^20^ on twisted graphene films grown by CVD on Ni and transferred on Au where the measured v_*f*_ was 0.87 × 10^6^ m/s for a twist angle of 3.5°. On the contrary, using similar LLS experiments on a different system, graphene on SiC C-face^22^, it was reported that the LL sequences were independent of the measured moiré periods for a wide range of twist angles. It was later argued that the large moiré period could be associated to a small twist angle between the first and third layer, both surrounding a second layer with a large relative twist^30^. This is not the case for the TBG grains measured here, since we know^26^ that the graphene thickness in these grains is limited to bilayers, thus eliminating the possible effect of additional graphene layers. Other experimental works that demonstrated renormalization involved CVD-grown or exfoliated graphene monolayers transferred on graphite^19^ or few layer graphene on HOPG^21^. These conflicting results on *v*_*f*_ renormalization could be linked to the substrate material through its electronic structure, if the interaction between the substrate and the first layer could affect the interlayer coupling between twisted graphene layers. Indeed, following a LLS study on naturally decoupled TBG on HOPG, it was concluded that the interlayer coupling plays a significant role on *v*_*f*_ renormalization^21^. This dependence on the interlayer coupling parameter was theoretically calculated^12^. The renormalization issue should be addressed based on these factors in order to resolve the current theoretical and experimental discrepancies. In this work, nanoARPES proved to be a powerful tool to study the electronic properties of single TBG grains. Future nanoARPES experiments to investigate the presence of van Hove singularities as concluded in earlier studies^14^,^15^ for small twist angles should be undertaken, i.e. performing detailed nanoARPES measurements along the K-K′ direction.

## Methods

The graphene samples were grown on *n*-type 4H:SiC(000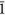) using Si flux-assisted molecular beam epitaxy (MBE) at a temperature of ~1280 °C under ultra-high vaccuum (UHV) conditions^37^,^38^,^39^,^40^. A high-flux Si effusion cell was utilized to compensate the Si sublimation thereby avoiding graphitization during high temperature MBE (above ~1100 °C). It leads to graphene on the C-face of SiC with an identical stacking structure as the one obtained by graphitization, although with a better thickness control and comparatively larger grains at low thickness^40^. Two sets of samples were produced under identical growth conditions in the MBE chamber, and then transferred under air either for ARPES or for STM experiments. Just after transfer, each sample was annealed for a few hours at ~950 °C under UHV.

The STM studies were performed with a UHV Omicron NanoTechnology system. All images were recorded at room temperature in the constant-current mode using a W tip. The nanoARPES experiments were carried out at the ANTARES beamline at the SOLEIL synchrotron^33^,^34^,^35^,^36^,^37^,^38^,^39^,^40^,^41^. The incident photon beam is focused to a ~120 nm spot size. The E(k) dispersion measurements were obtained at a sample temperature of ~100 K, using a hemispherical analyzer Scienta R4000 with respective energy and momentum resolutions of 5 meV and 0.005 Å^−1^. NanoARPES intensity real-space maps are obtained by recording the E(k) dispersion at a fixed energy mode using an acceptance angle of 14° for a short integration time at each point in the scanned area, and then integrating the photoelectron intensity over E and k in the selected range.

## Additional Information

**How to cite this article**: Razado-Colambo, I. *et al*. NanoARPES of twisted bilayer graphene on SiC: absence of velocity renormalization for small angles. *Sci. Rep.* **6**, 27261; doi: 10.1038/srep27261 (2016).

## Supplementary Material

Supplementary Information
