# Supplementary material for: NanoARPES of twisted bilayer graphene on SiC: absence of velocity renormalization for small angles
Source: Sci Rep. 2016 Jun 6;6:27261. doi: 10.1038/srep27261 (PMC4893698; doi:10.1038/srep27261)
Supplement: Supplementary Information [file srep27261-s1.doc]

**Supplementary information: NanoARPES of twisted bilayer graphene on SiC: absence of velocity renormalization for small angles**

I. Razado-Colambo^1^, J. Avila^2^, J.-P. Nys^1^, C. Chen^2^, X. Wallart^1^, M.-C. Asensio^2^ and

D. Vignaud^1*^

^1^ I.E.M.N., UMR CNRS 8520, Av. Poincaré CS 60069, 59652 Villeneuve d'Ascq Cedex, France

^2^ Synchrotron SOLEIL, L'Orme des Merisiers, Saint Aubin-BP 48, 91192 Gif sur Yvette Cedex, France

* corresponding author : Dominique.Vignaud@univ-lille1.fr

**1. AB stacked multilayer graphene domain nanoARPES**

**Figure S1:** A typical nanoARPES E(k) dispersion corresponding to an AB-stacked multi-layer domain (along the Γ-K direction of one N-oriented grain, measured at 100 eV photon energy).

**2. Relative twist angle determination and nanoARPES limitations**

When both linear bands are not exactly aligned along the detector azimuth (e.g. the bands shown in Fig. 3g), the nanoARPES experiments do not allow to determine unambiguously the relative positions of the bands. This point is illustrated by the scheme in Fig. S2, showing three linear bands twisted relative to the detector azimuth depicted by the vertical dashed line. The two blue bands present the same energy shift ΔE_1_ (corresponding to ΔK_1_ and a twist angle θ_1_) compared to the virtual domain aligned with the detector. Compared to the red band, characterized by ΔE_2_ and ΔK_2_, the total momentum shift is ΔK_2_ ± ΔK_1_, corresponding to a twist angle θ_2_ ± θ_1_.

**Figure S2:** Scheme illustrating the case of three twisted graphene layers, two of them being symmetrically twisted with regards to the detector azimuth (shown as a vertical dashed line). The ARPES measurement plane E(k) is perpendicular to the figure plane, going through the vertical dashed line.

The twist angle in TBG is determined from the measured energy difference ΔE between the maxima of the valence band (see Fig. 3b), using equations 2 and 3. When a 1.5 eV range below the Fermi level is scanned, the Dirac point lying 0.2 eV below the Fermi level, the experimentally measured energy difference ΔE could not exceed 1.3 eV. This corresponds to *θ*=6°, if there is no velocity renormalization. Because a band cannot be properly identified if it is not detected over an estimated range ΔE~0.4 eV, this further restricts the maximum twist angle to *θ*~4°. For example, the spectra shown in Fig. S3 is an example of a single band spectra. It could be associated either with a TBG domain with a large twist angle (>4°), or with a monolayer domain.


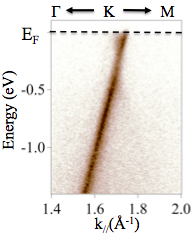


**Figure S3:** A typical single band nanoARPES E(k) dispersion recorded for a W oriented graphene domain (along the Γ-K direction of the W grain at 100 eV photon energy).
